# Identification of *Toxoplasma gondii* cAMP Dependent Protein Kinase and Its Role in the Tachyzoite Growth

**DOI:** 10.1371/journal.pone.0022492

**Published:** 2011-07-20

**Authors:** Hitomi Kurokawa, Kentaro Kato, Tatsuya Iwanaga, Tatsuki Sugi, Atsushi Sudo, Kyousuke Kobayashi, Haiyan Gong, Hitoshi Takemae, Frances C. Recuenco, Taisuke Horimoto, Hiroomi Akashi

**Affiliations:** 1 Department of Veterinary Microbiology, Faculty of Agriculture, The University of Tokyo, Bunkyo-ku, Tokyo, Japan; 2 School of Medicine, Kobe University, Chuo-ku, Kobe, Japan; Weill Cornell Medical College, United States of America

## Abstract

**Background:**

cAMP-dependent protein kinase (PKA) has been implicated in the asexual stage of the *Toxoplasma gondii* life cycle through assaying the effect of a PKA-specific inhibitor on its growth rate. Since inhibition of the host cell PKA cannot be ruled out, a more precise evaluation of the role of PKA, as well as characterization of the kinase itself, is necessary.

**Methodology/Principal Finding:**

The inhibitory effects of two PKA inhibitors, H89, an ATP-competitive chemical inhibitor, and PKI, a substrate-competitive mammalian natural peptide inhibitor, were estimated. In the *in vitro* kinase assay, the inhibitory effect of PKI on a recombinant *T. gondii* PKA catalytic subunit (TgPKA-C) was weaker compared to that on mammalian PKA-C. In a tachyzoite growth assay, PKI had little effect on the growth of tachyzoites, whereas H89 strongly inhibited it. Moreover, *T. gondii* PKA regulatory subunit (TgPKA-R)-overexpressing tachyzoites showed a significant growth defect.

**Conclusions/Significance:**

Our data suggest that PKA plays an important role in the growth of tachyzoites, and the inhibitory effect of substrate-competitive inhibitor PKI on *T. gondii* PKA was low compared to that of the ATP competitive inhibitor H89.

## Introduction

*Toxoplasma gondii* is an obligate intracellular apicomplexan parasite that is an important pathogen of humans and animals. *T. gondii* causes encephalitis in immunocompromised patients, and progressive encephalitis in children infected in utero [1], [2]. The life cycle of *T. gondii* consists of two phases: the sexual, which takes place only in felines, and the asexual, which takes place in all mammalian and avian hosts [3]. In the asexual phase, the parasite switches between two different developmental forms. The tachyzoite is the rapidly growing form of the parasite and is responsible for the infection and toxoplasmosis. Tachyzoites multiply asexually, invade host cells, and are distributed via the blood stream and lymphatic system throughout the body. In healthy animals, the infection is normally controlled by the immune system. After being triggered by the immune system, tachyzoites differentiate into slow-growing, encysted bradyzoites, which reside in the central nervous system and muscle tissue for the life of the host, hidden from the immune system [4]. In immunocompromised patients, such as those with human immunodeficiency virus (HIV) infection, bradyzoites can reactivate and differentiate into tachyzoites, leading to a severe toxoplasmosis [5]. Although drugs for treatment of toxoplasmosis are available, they are poorly tolerated, have severe side effects, and cannot act against chronic *Toxoplasma* infections [6], [7]. Therefore, new anti-*T. gondii* drugs are urgently needed. Studies on the basic biology of this organism are thus necessary for discovery of novel targets, and may also serve as a model system for the study of other apicomplexan parasites.

Eukaryotic signaling pathways regulate a spectrum of intracellular activities; for example, the cAMP-dependent pathway is known to influence gene expression, apoptosis, tissue differentiation, and cellular proliferation [8]. The main enzymatic component of this signaling pathway is cAMP-dependent protein kinase (PKA). In its non-active form, PKA is a tetramer composed of two catalytic subunits (PKA-C) and two regulatory subunits (PKA-R). Binding of cAMP to PKA-R, each subunit of which contains two cAMP-binding sites, releases the PKA-C subunits, resulting in their activation [9]. In addition to the PKA-R subunits, PKA-C activity is regulated through the binding of its natural peptide inhibitor, protein kinase A inhibitor (PKI). PKI contains pseudosubstrate sites, which allows it to bind to PKA-C with high affinity and inhibit PKA-C activity by competing with its substrate [10].

Both cAMP and PKA have been shown to be essential signaling components in the life cycles of many eukaryotic pathogens. In *Plasmodium falciparum*, cAMP has been implicated in the differentiation of gametocytes [11], and PKA activity might be necessary for completion of the asexual cycle or reinvasion of erythrocytes [12]. A key role for PKA in regulating parasite differentiation and gene expression has also been indicated in *Leishmania major* [13], [14], *Giardia lamblia* [15], and *Schistosoma mansoni* [16], [17]. In *T. gondii*, studies using cAMP analogs and cyclic nucleotide phosphodiesterase (PDE) inhibitors, which inhibit inactivation of cAMPs, implicated cAMP signaling in the asexual stage of the parasite [18]. Recently, H89, a specific inhibitor of PKA, was reported to cause decreased bradyzoite differentiation and replication of tachyzoites [19]. Although these data indicate a possible role of cAMP and PKA in the asexual cycle of *T. gondii*, whether these effects are extracellular (i.e., host-mediated), intracellular (i.e., within the parasite), or due to stimulation of both host and parasite signaling pathways remains to be determined. Moreover, although H89 is considered to be a specific inhibitor of PKA, several recent reports have suggested that H89 inhibits other kinases such as mitogen- and stress-activated protein kinase 1, p70 ribosomal protein S6 kinase or Rho-dependent protein kinase II [20], [21], [22]. Therefore, H89 should not be the single source of evidence for PKA involvement.

In this study, we compared the inhibitory effects of ATP competitive chemical inhibitor H89 and mammalian natural peptide inhibitor PKI in an *in vitro* kinase assay using recombinant *T. gondii* PKA catalytic subunit (TgPKA-C), as well as tachyzoite growth assay. The effect of PKI was weaker in TgPKA-C compared to mammalian PKA-C in the *in vitro* kinase assay, and PKI did not inhibit tachyzoite growth. These data support the hypothesis that the inhibitory effect of H89 on tachyzoite growth is due to TgPKA-C inhibition, since the possible inhibition of host cell PKA-C activity by PKI did not result in the inhibition of tachyzoite growth. Moreover, we generated a parasite line that expressed TgPKA-R stably, in which inhibiting the activity of parasite PKA without any effect on host cell PKA might be possible. These parasites also showed decreased growth. According to these data, TgPKA-C does indeed play an important role in the asexual stage of the *T. gondii* life cycle.

## Results

### Identification of TgPKA-C

**Figure 1:**
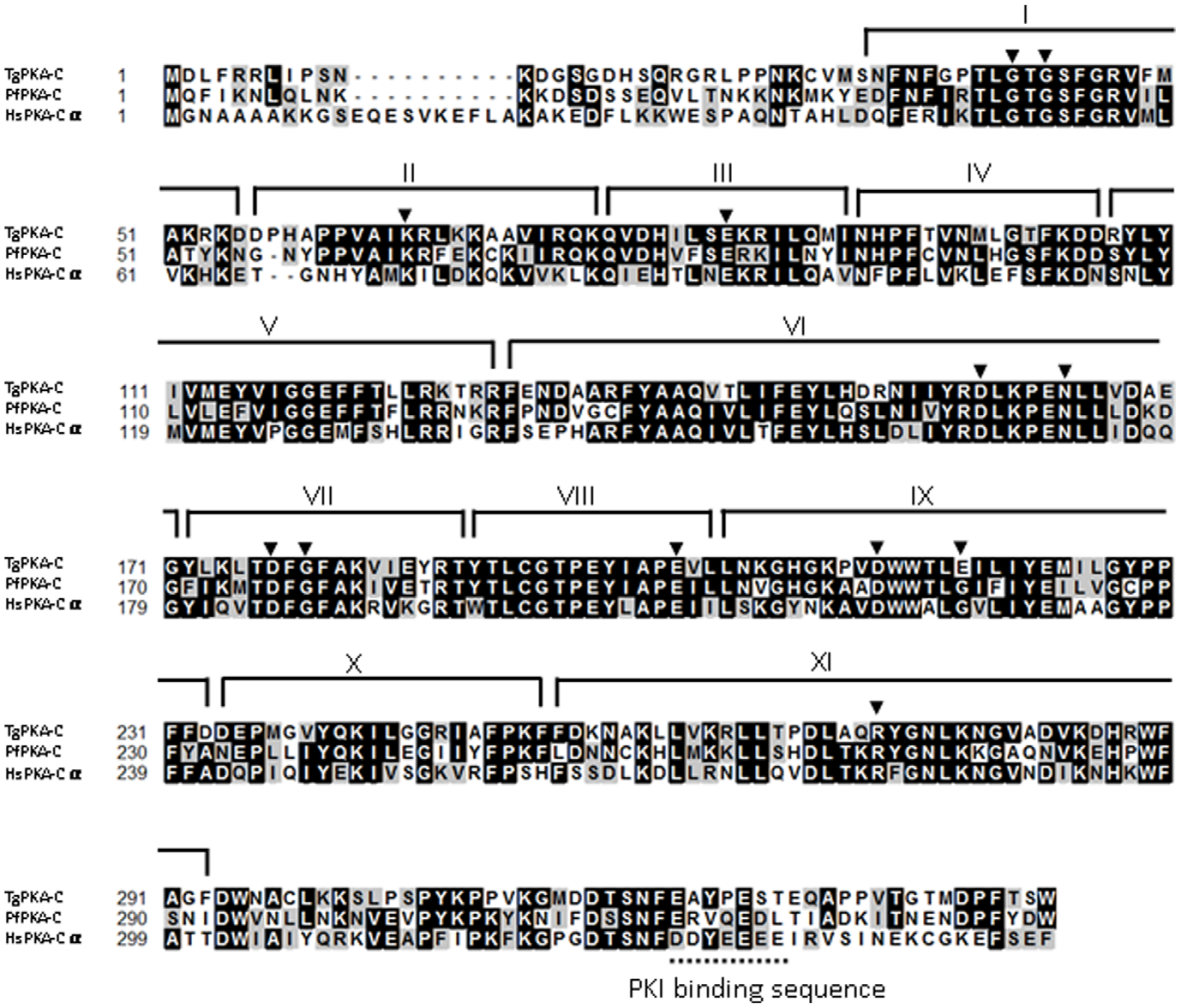
Identification of TgPKA-C. Comparison of predicted TgPKA-C amino acid sequences (ToxoDB identifier; TGGT1_081170) with those of *P. falciparum* PKA-C (PfPKA-C, GenBank Accession Number: AAB70118) and *H. sapiens* PKA-Cα (HsPKA-Cα, GenBank Accession Number: NP_002721). Amino acid identity (black boxes) and similarity (gray boxes) are shown within the protein kinase domain. Gaps introduced to optimize alignments are marked by dashes. Roman numerals indicate the 11 conserved protein kinase subdomains [23]. The 12 most highly conserved residues are highlighted with filled triangles. The sequence thought to be necessary for binding to mammalian PKI is shown with a dotted line.

The amino acid sequence alignment of the putative TgPKA-C (ToxoDB identifier; TGGT1_081170), which we identified for the first time, is shown in Figure 1 together with those of *P. falciparum* and *Homo sapiens*. The putative TgPKA-C (343 amino acid residues) showed 54% identity with *H. sapiens* PKA-Cα (HsPKA-Cα, GenBank Accession Number: NP_002721) and 57% identity with *P. falciparum* PKA-C (PfPKA-C, GenBank Accession Number: AAB70118). A multiple alignment using the ClustalW program showed that the 11 major subdomains of protein kinases (I–XI) [23] are conserved in the amino acid sequence of TgPKA-C. Moreover, highly conserved individual amino acids that are involved in ATP binding, peptide binding, stabilizing, or autophosphorylation were also seen in TgPKA-C amino acid sequences [24], [25].

### Identification of TgPKA-R

**Figure 2:**
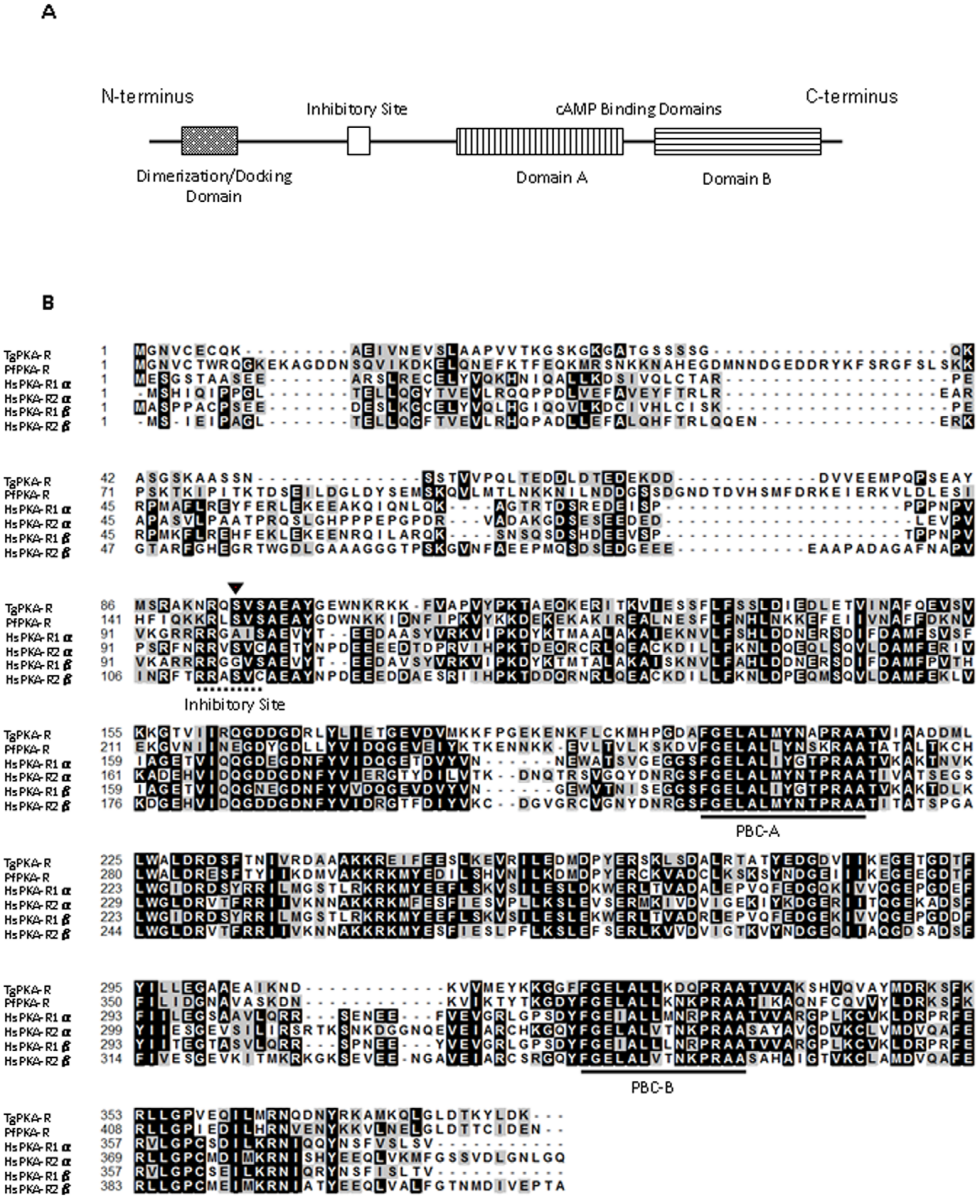
Identification of TgPKA-R. **A**, Schematic linear domain structure of a mammalian PKA-R. The N-terminal dimerization domain, inhibitory site, cAMP-binding domain A, and cAMP-binding domain B are shown. **B**, Comparison of predicted TgPKA-R amino acid sequences (ToxoDB identifier: TGGT1_048350) with those of *P. falciparum* PKA-R (PfPKA-R, GenBank Accession Number: AJ441326) and *H. sapiens* PKA-R isoforms Iα (HsPKA-RIα, GenBank Accession Number: NP_002725), Iβ (GenBank Accession Number: NP_001158230), IIα (GenBank Accession Number: NP_004148), and IIβ (GenBank Accession Number: NP_002727). Amino acid identity (black boxes) and similarity (gray boxes) are shown within the protein kinase domain. Gaps introduced to optimize alignments are marked by dashes. The conserved phosphate-binding cassettes of cAMP-binding domains A (PBC-A) and B (PBC-B) are shown with a solid line, and the inhibitory sequence is indicated by a dotted line. The phosphorylable serine is indicated by a black arrow.

Figure 2A shows the general domain structure of a mammalian PKA-R. PKA-R is an multi-domain protein composed of a dimerization/docking (D/D) domain at the N terminus [the docking site for A-kinase anchor proteins (AKAPs)], followed by an inhibitory site (for binding PKA-C) and two cAMP-binding domains, A and B [26]. An amino acid sequence alignment of the predicted TgPKA-R with those of *P. falciparum* and *H. sapiens*, is shown in Figure 2B. The predicted TgPKA-R (ToxoDB identifier: TGGT1_048350) (386 amino acid residues) showed 37% identity with *H. sapiens* PKA regulatory subunit Iα (HsPKA-RIα, GenBank Accession Number: NP_002725), 35% identity with HsPKA-RIIα (GenBank Accession Number: NP_004148), 36% identity with HsPKA-RIβ (GenBank Accession Number: NP_001158230), and 32% identity with HsPKA-RIIβ (GenBank Accession Number: NP_002727), as well as 52% identity with *P. falciparum* PKA-R (PfPKA-R, GenBank Accession Number: AJ441326). TgPKA-R possessed the two most conserved regions of PKA-R: the phosphate-binding cassettes (PBCs) of cAMP-binding domains A and B, specific to proteins of the PKA-R family [27]. The TgPKA-R amino acid sequence also harbored a site of inhibition, which contained a phosphorylatable serine, a feature characteristic of the RII subunit of mammalian PKA. In contrast, a D/D domain was not detected in TgPKA-R.

### Expression and purification of TgPKA-C in the wheat germ cell-free protein synthesis system

**Figure 3:**
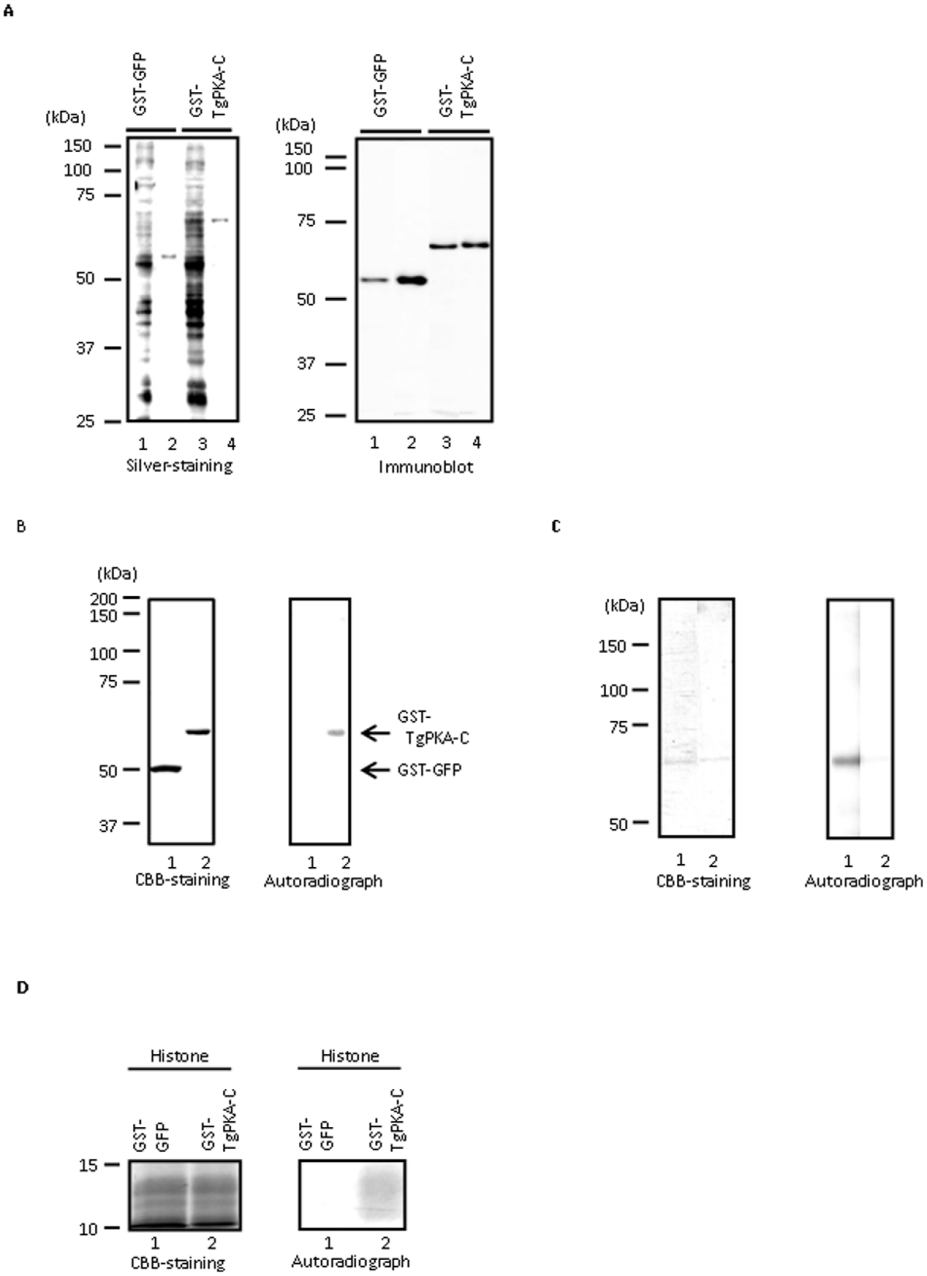
Expression, purification and kinase activity of TgPKA-C. **A**, A silver-stained ge and an immunoblot of purified GST-GFP or GST-TgPKA-C generated in the wheat germ cell-free protein synthesis system using pEU-GST-GFP (lanes 1 and 2) or pEU-GST-TgPKA-C (lanes 3 and 4). Total wheat germ extracts (lanes 1 and 3) were subjected to affinity chromatography on glutathione-Sepharose beads (lanes 2 and 4). The proteins were separated on denaturing gels and subjected to silver staining or transferred onto a nitrocellulose sheet and reacted with the anti-GST antibody. Molecular masses (kDa) are shown on the left. **B**, Purified GST-GFP (lane 1) or GST-TgPKA-C (lane 2) was incubated in kinase buffer containing [γ-^32^P]ATP, separated on a denaturing gel, and Coomassie stained (left panel). Autoradiograph of the gel (right panel). Arrows indicate the migration of GST-TgPKA-C and GST-GFP. **C**, Purified GST-TgPKA-C was incubated in kinase buffer (lane 1). The labeled protein was treated with λ-protein phosphatase (lane 2). Reaction mixtures were then subjected to resolution on an 8% SDS-PAGE gel, followed by Coomassie staining (left panel). Autoradiograph of the gel (**C**). **D**, Purified GST-GFP (lane 1) or GST-TgPKA-C (lane 2) was incubated in kinase buffer with Histone II_AS_ and separated on a 15% denaturing gel followed by Coomassie staining (left panel). Autoradiograph of the gel (right panel). Molecular masses (kDa) are shown on the left.

To characterize the function of the protein, we expressed and purified TgPKA-C as a GST fusion protein using a wheat germ cell-free protein synthesis system. Purified protein was then electrophoretically separated in a denaturing gel and either silver-stained or immunoblotted with rabbit antiserum containing α-GST antibody (Figure 3A). The purified proteins from the wheat germ extracts after cell-free protein synthesis with either pEU-GST-GFP [for the expression of GST and green fluorescence protein (GFP) fusion protein] or pEU-GST-TgPKA-C contained one major purified protein with an M_r_ of 53,000 or 69,000, respectively, as detected by silver staining. These proteins reacted with antiserum containing α-GST antibody. These data indicate that we successfully purified the desired GST fusion proteins.

### Protein kinase activity of purified TgPKA-C

Many protein kinases possess autophosphorylating activity [28]. Autophosphorylation of PKA-C is necessary for its enzymatic activation [29] and interaction with PKA-R [30]. We thus used a kinase assay to determine whether purified TgPKA-C did in fact possess such activity. Purified GST–GFP fusion protein, which was incubated in kinase buffer and electrophoretically separated, did not contain any labeled bands (Figure 3B, lane 1). However, in the autoradiographic image of purified TgPKA-C, a protein band with an apparent M_r_ of 69 000 was labeled (Figure 3B, lane 2). The electrophoretic mobility of labeled TgPKA-C was the same as that of purified TgPKA-C stained with Coomassie brilliant blue (CBB; Figure 3B). To determine if the labeling of TgPKA-C with [γ-^32^P]ATP was due to phosphorylation, labeled TgPKA-C was boiled to inactivate kinases and incubated with λ-protein phosphatase (New England Biolabs, Ipswich, MA) in accordance with the manufacturer's instructions. The labeled TgPKA-C band was eliminated by phosphatase treatment, indicating that TgPKA-C was labeled with [γ-^32^P]ATP via phosphorylation (Figure 3C). These data suggested that TgPKA-C levels remained relatively unchanged after boiling, and so boiling did not result in loss of TgPKA-C. Thus, TgPKA-C phosphorylates itself and indeed possesses protein kinase activity. Next, we investigated whether TgPKA-C has the ability to phosphorylate other substrate(s). When histone II_AS_ was used as a representative substrate for protein kinase, it was labeled by TgPKA-C (Figure 3D, lane 2), but not by GFP (Figure 3D, lane 1). The expression of each GST fusion protein and histone II_AS_ and the identity of the radiolabeled bands were verified by CBB staining (Figure 3B and D). A positive reaction was seen only in the case of histone II_AS_ phosphorylated by TgPKA-C (Figure 3D, lane 2) as no protein contamination was detected in the migration range of histone II_AS_ in the lane of purified GFP (Figure 3D, lane 1). These results indicate that TgPKA-C possesses the ability to phosphorylate both itself and other substrate(s).

### Susceptibility of recombinant TgPKA-C to PKA-specific inhibitors

**Figure 4:**
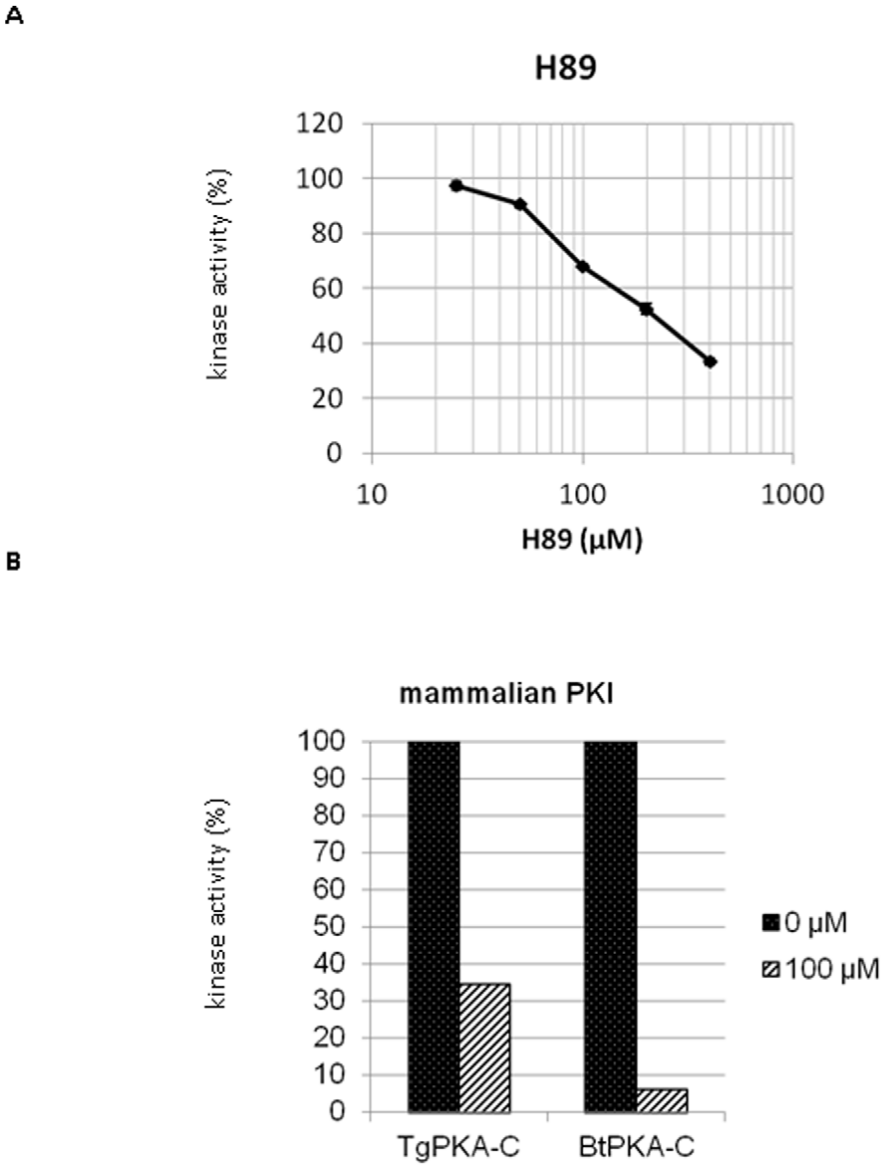
Effects of mammalian PKA inhibitors on the kinase activity of TgPKA-C *in vitro.* **A** and **B**, Kinase activities of TgPKA-C and BtPKA-C were calculated in the presence of 400, 200, 100, 50 and 25 *µ*M of H89 (**A**) or 100 *µ*M of bovine PKI (**B**). Reactions were carried out at 30°C for 30 min and terminated by boiling in SDS-PAGE sample buffer. Reaction mixtures were then subjected to resolution by 15% SDS-PAGE followed by Coomassie staining. Phosphate incorporation into Histone II_AS_ was measured by scintillation counting of excised gel fragments. Enzyme activity in the absence of inhibitors was taken to be 100%. Representative results of three independent determinations are shown.

An inhibition assay was used to estimate the effect of PKA specific inhibitors on TgPKA-C. As shown in Figure 4A, inhibition assays of TgPKA-C by H89 was performed on the concentrations of 400, 200, 100, 50 and 25 *µ*M. The estimated IC_50_ was 175 *µ*M, which might be much higher than that of mammal PKA [31]. Inhibitory assay of PKI was performed in the presence or absence of 100 *µ*M PKI_5-24_. In addition to TgPKA-C, *Bos taurus* PKA-C (BtPKA-C; Promega, Madison, WI) was included as a positive control. Compared to BtPKA-C, inhibition by PKI was much weaker in TgPKA-C (Figure 4B). These data indicate that susceptibility of TgPKA-C on both inhibitors differs from that of mammalian PKA-C.

### Effect of PKA-C inhibitors on the growth of parasites

**Figure 5:**
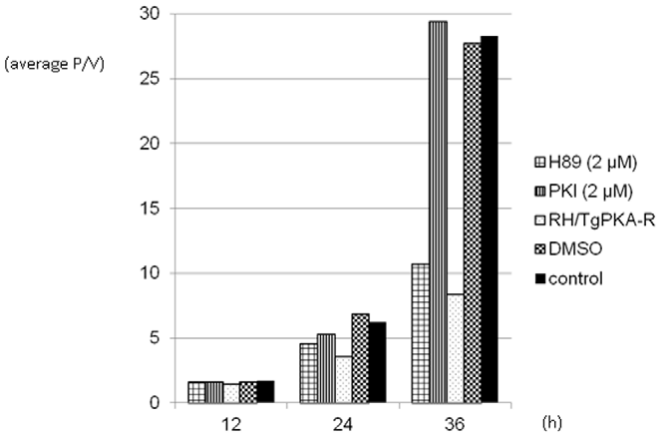
*T. gondii* tachyzoite growth assay. Average parasite number per parasitophorous vacuole at 12, 24, and 36 h postinfection. RH/PKA-R or RH/GFP parasites were added to Vero cell monolayers at a MOI of 1∶10 and incubated for 2 h at 37°C. Extracellular parasites were then removed by washing three times with PBS(–) and returned to culture in complete medium with or without PKA inhibitor. The final concentration of inhibitors H89 (Promega) and PKI_14-22_ (Calbiochem) was 2 *µ*M. P/V: parasites per vacuole. Representative results of three independent determinations are shown.

To investigate the role of TgPKA in the asexual stage, we performed a tachyzoite growth assay in the presence of H89 and PKI. Tachyzoites treated with H89 have been reported to show an increased doubling time [19], indicating a potential role of TgPKA in replication. However, an effect of H89 on host cell PKA still cannot be ruled out, since mammalian PKA showed high susceptibility to H89 in the *in vitro* kinase assay (Figure 4B). To clarify whether the growth defect was due to inhibition of *T. gondii* or host cell PKA, we compared the effect of H89 with that of PKI in a tachyzoite growth assay. Since PKI_5–24_, which we used in the *in vitro* kinase assay, is not cell-permeable, we used the cell-permeable, myristolated PKI_14–22_. The final concentration of both H89 [19] and PKI was 2 *µ*M. Figure 5 shows the average parasite number per parasitophorous vacuole (P/V) at 12, 24, and 36 h post-invasion. Vacuoles containing more than 32 parasites were calculated as 32 P/V, since above this value discriminating individual parasites became problematic. The average P/V in controls was 1.71, 6.22, and 28.3 at each time point. In dimethyl sulfoxide (DMSO)-treated parasites (a vehicle of H89), the average P/V was 1.64, 6.90, and 27.7 at each time point, similar to those of the control parasites. The average P/V of H89-treated parasites was markedly lower (1.63, 4.61, and 10.7). The growth rate of H89-treated parasites at 36 h was about half that of the control, suggesting growth inhibition. This is consistent with previous reports [19]. In contrast, in mammalian PKI-treated parasites, the average P/V values were 1.62, 5.35, and 29.4. Since no significant difference between the P/V of the control and PKI-treated parasites was observed, PKI seems to have no inhibitory effect on tachyzoite growth. This result indicates that the susceptibility of TgPKA-C to PKI is lower than that to H89.

### Interaction of recombinant TgPKA-C and TgPKA-R *in vitro*

**Figure 6:**
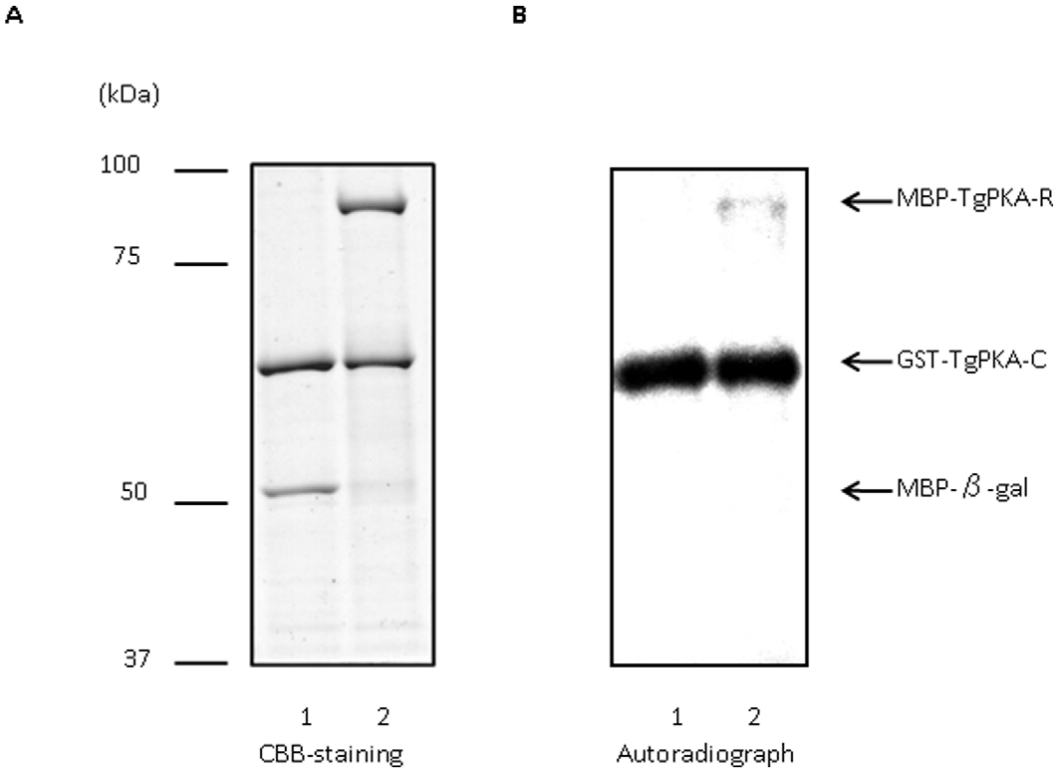
Interaction of GST-TgPKA-C and MBP-TgPKA-R. **A**, Purified MBP-β-gal (lane 1) or MBP-TgPKA-R (lane 2) was incubated with GST-TgPKA-C in kinase buffer containing [γ-^32^P]ATP, separated on a denaturing gel, and Coomassie stained. **B**, Autoradiograph of the gel shown in (**A**). Arrows indicate the migration of GST-TgPKA-C, MBP-β-gal, or MBP-TgPKA-R. Molecular masses (kDa) are shown on the left.

To determine whether TgPKA-R interacts with TgPKA-C *in vitro*, we used TgPKA-C and recombinant TgPKA-R in an *in vitro* kinase assay. We first expressed TgPKA-R as an MBP fusion protein. Next, we incubated purified MBP-TgPKA-R with GST-TgPKA-C. MBP-β-gal-α was used as a negative control. As shown in Figure 6A, lane 1, MBP-β-gal-α did not show evidence of a positive reaction. However, in the autoradiographic image of purified MBP-TgPKA-R, a protein band with an apparent Mr of 86,000 was labeled (Figure 6B, lane 2). These data demonstrate that TgPKA-C phosphorylates TgPKA-R *in vitro*.

### Growth assay of TgPKA-R-overexpressing parasites

**Figure 7:**
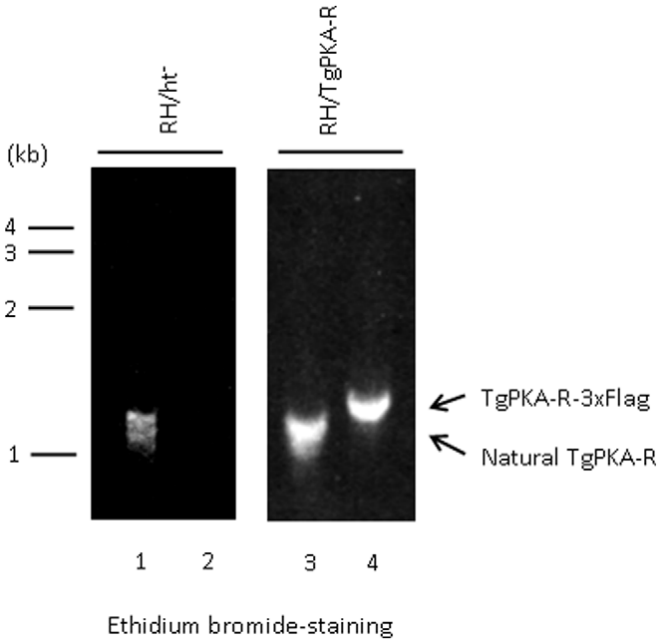
Expression of TgPKA-R-3xFlag mRNA in RH/PKA-R. Confirmation of the expression of TgPKA-R-3xFlag mRNA in RHPKA-R by RT-PCR. Total RNAs extracted from RH/ht^−^ or RH/PKA-R were used as templates. PCR samples were resolved on a 1.5% agarose gel and stained with ethidium bromide.

To clarify the role of TgPKA in the asexual stage of the *T. gondii* life cycle, inhibiting its kinase activity without affecting the host cell is necessary. We hypothesized that an excess of PKA regulatory subunits may inhibit the activity of catalytic subunits. Therefore, we generated a *T. gondii* line that overexpresses TgPKA-R. The derived line was designated RH/TgPKA-R. RH/TgPKA-R expressed TgPKA-R-3xFlag mRNA. Total RNA extracted from RH/TgPKA-R contained an mRNA of approximately 1.3 kbp (Figure 7, lane 4), as detected by RT-PCR using the primers 5′-CGAATTCGGGTAACGTTTGTGAATGCC-3′ and 5′-CGCGTTAATTAACTACTTGTCATCGTCATCC-3′, whereas no band was detected from total RNA extracted from the parental strain RH/ht^−^ (Figure 7, lane 2). When a growth assay was performed using this RH/TgPKA-R line, a significant defect in growth was observed. The average parasite numbers per vacuole were 1.46, 3.60, and 8.39 at 12, 24, and 36 h post-invasion, respectively (Figure 5). Since the host cell PKA was not inhibited, one may reasonably assume that the decreased parasite growth rate was a result of TgPKA-C inhibition.

## Discussion

We identified *T. gondii* PKA-C and PKA-R, which possess the highly conserved domains in PKA-Cs or PKA-Rs of *H. sapiens* and *P. falciparum*. GST-TgPKA-C was successfully expressed and purified using a wheat germ cell-free protein synthesis system. We also demonstrated that purified TgPKA-C possesses both auto and substrate kinase activity.

We determined the effect of two PKA specific inhibitors, H89 and PKI, on TgPKA-C activity. The IC_50_ of H89 on TgPKA-C was 175 *µ*M, while that of bovine PKA-C has been reported to be approximately 50 nM [31]. Such a difference in the IC_50_s might be due to the expression system, since TgPKA-C was expressed using the germ cell-free protein synthesis system while BtPKA-C we used was native. Using PKI, difference in the susceptibility could be also observed. Our data suggested that inhibition of TgPKA-C by PKI was weak compared to mammalian PKA-C. This is consistent with our data from *P. falciparum* [32]. We also estimated the effect of H89 and mammalian PKI on tachyzoite growth. Whereas H89 reduced the growth of tachyzoites, as previously reported [19], PKI at the same concentration had no, or at high concentrations, a vastly reduced effect (data not shown). These data, together with those of the *in vitro* kinase assay, show that the susceptibility of TgPKA-C to mammalian PKI was low compared to mammalian PKA-C. Since host PKA is likely strongly inhibited by mammalian PKI, one may reasonably consider that host PKA inhibition has no significant effect on *T. gondii* growth, at least *in vitro*. Therefore, the effect of H89 on growth appears to have been due to inhibition of *T. gondii* PKA-C, suggesting an important role for PKA-C in the growth of tachyzoites.

The low susceptibility of TgPKA-C to PKI might be due to its lack of the PKI binding sequence “DDYEEEE,” which lies near the C-terminus of PKA-C (Figure 1). Lack of such a binding sequence occurs in other apicomplexan parasites, e.g., *P. falciparum* [33], *Babesia bovis*, *Theileria annulata*, and *Cryptosporidium parvum*. Natural PKI in mammalian cells regulates free PKA-C not only through its kinase activity but also by localization, such as exporting it from the nucleus back to the cytoplasm [10], [34], [35]. Therefore, the lack of the PKI-binding sequence in PKA-C suggests the existence of a different regulatory mechanism in apicomplexan parasites. This difference in PKA-C regulation between the apicomplexan parasites and their hosts renders PKA-C a potential target for novel therapeutics.

Furthermore, we succeeded in constructing TgPKA-R-overexpressing parasites. A significant defect in the growth of these parasites was detected. This suggests strongly that inhibition of *T. gondii*, but not host cell, PKA-C was the cause of the growth inhibition. Thus, these data support the hypothesis that *T. gondii* PKA-C plays a significant role in tachyzoite replication.

The mechanism by which TgPKA-R, overexpressed in transgenic parasites, inhibited the activity of natural PKA-C is unclear. Two possibilities exist: TgPKA-R may act as a pseudosubstrate of TgPKA-C by inhibiting its binding to a substrate, or it may act as a cAMP sink, thereby reducing available cAMP so that PKA-C cannot be dissociated from PKA-R. When we used GST-TgPKA-C and MBP-TgPKA-R under cAMP-free conditions in an *in vitro* kinase assay, no reduction in its phosphorylation ability was observed (data not shown). However, in the experiment in which *P. falciparum* lysate containing cAMPs was used instead of recombinant TgPKA-C, phosphorylation of PfPKA-C markedly decreased [36]. Although other factors should be considered, the presence of cAMP may be the reason for this difference. Therefore, the presence of elevated TgPKA-R levels may have inhibited the activity of TgPKA-C by reducing available cAMP.

Further understanding of the biology of TgPKA may best be gained by investigation of the pathways and proteins with which TgPKA-C interacts. Since PKA is known to be involved in various activities of mammalian cells, many types of substrate may also be present in *T. gondii*. Several proteins have recently been reported to interact with PKA-C in apicomplexan parasites. In *P. falciparum*, the merozoite transmembrane protein AMA1, which is involved in the invasion of erythrocytes, has been reported to be phosphorylated by PKA [37]. Since *T. gondii* possesses a homolog of this protein (TgAMA1), TgPKA may also interact with it, thereby regulating invasion. In *Trypanosoma cruzi*, yeast two-hybrid experiments revealed that several proteins with known functions interact with PKA [38], [39]. These potential substrates are presumably important for the regulation of *T. cruzi* growth, adaptation, and differentiation. Since some of these proteins, for example, hexokinase and aquapoline, also exist in *T. gondii*, investigating their interaction(s) with TgPKA-C might be interesting.

## Materials and Methods

### Cells and parasites

Tachyzoites of *T. gondii* RH strain and RH/ht^−^ strain (kindly provided by Dr. X. Xuan) were used in this study. Parasites were maintained in monolayers of Vero cells cultured in Dulbecco's modified Eagle's medium (DMEM) that contained 7.5% fetal calf serum (FCS), 2 mM l-glutamine, 20 mM HEPES (pH 7.5), streptomycin, and penicillin. Host Vero cells were passaged in the same medium.

### Plasmids

*T. gondii* RH strain mRNA was isolated from infected Vero cells using the TRIZOL reagent (Invitrogen, Carlsbad, CA) according to the manufacturer's instructions. The entire TgPKA-C open reading frame (ORF) was amplified by RT-PCR using parasite mRNA as the template and the following primers: forward, 5′-CCGGCTCGAGGATCTTTTTCGCCGTCTAAT-3′ and reverse, 5′-CGGGATCCTTACCACGACGTGAAAGGGT-3′. The PCR product was digested with *Xho*I/*Bam*HI and cloned into the *Xho*I and *Bam*HI sites of pEU (CellFree Sciences, Yokohama, Japan) to express a glutathione S-transferase (GST) fusion protein. The resultant plasmid was designated pEU-GST-TgPKA-C. The entire TgPKA-R ORF was amplified from parasite mRNA by RT-PCR using the primers 5′-CGAATTCGGTAACGTTTGTGAATGCCAAAAG-3′ and 5′-GGGCTGCAGTTATTTGTCGAGGTATTTGGTGTCG-3′. Amplified fragments were digested with *Eco*RI/*Pst*I and cloned into the *Eco*RI and *Pst*I sites of pMal-c (New England BioLabs, Beverly, MA) in-frame with maltose-binding protein (MBP), to generate pMal-TgPKA-R. For stable expression, pMFH-TgPKA-R was generated as follows. The TgPKA-R ORF attached to the *Eco*RI/*Eco*RV restriction site was amplified from pMal-TgPKA-R using the primers 5′-CGAATTCGGGTAACGTTTGTGAATGCC-3′ and 5′-GGATATCTTTGTCGAGGTATTTGGTGTCG-3′. Amplified fragments were digested with *Eco*RI/*Eco*RV and cloned into the *Eco*RI and *Eco*RV sites of pMini.3×Flag.ht [40].

### Wheat germ cell-free protein synthesis system

Protein expression using the Wheat germ cell-free protein synthesis system (CellFree Sciences) was performed as described previously [41], [42]. Briefly, at the transcription step, 2 *µ*g of pEU-GST-GFP or pEU-GST-TgPKA-C was mixed with 18 *µ*L transcription mixture (transcription buffer with 2.5 mM NTP mix, 1 U/*µ*L RNase inhibitor, 1 U/*µ*L SP6 RNA polymerase; CellFree Sciences) and incubated at 37°C for 6 h. Each mRNA generated was then mixed with 10.8 *µ*L WEPRO1240G (CellFree Sciences) and 40 ng/*µ*L creatine kinase (Roche, Mannheim, Germany), then transferred to the bottom of the SUB-AMIX (CellFree Sciences) to form a bilayer and incubated at 16°C for 20 h.

### Purification of recombinant proteins

Wheat germ extracts were mixed with 15 *µ*L of a 50% slurry of glutathione sepharose beads (GE Healthcare, Little Chalfont, Buckinghamshire, UK) for 16 h. The beads were then washed three times with phosphate-buffered saline (PBS)(–). Purified protein captured on the beads was separated by 10% SDS-PAGE after boiling and either silver-stained or immunoblotted (Figure 3A) with α-GST antibody (Sigma-Aldrich, St. Louis, MO). Purification of recombinant protein expressed in *Escherichia coli* DH5α transformed with plasmids harboring MBP-TgPKA-R was performed according to the manufacturer's instructions. Briefly, bacterial cells cultured with 0.1 mM isopropyl β-d-1-thiogalactopyranoside (IPTG) at 37°C for 2 h were pelleted and solubilized in 1% Tween 20/PBS(–) using one freeze–thaw cycle and three 10 s bursts of ultrasonication. After centrifugation (10,500 rpm, 4°C, 10 min), the supernatant was mixed with amylose resin beads (New England BioLabs) at 4°C for 2 h, and the beads washed five times with 1% Tween 20/PBS(–).

### *In vitro* kinase assay

Purified GST-GFP or GST-TgPKA-C captured on glutathione-Sepharose beads was rinsed twice with washing buffer [50 mM Tris–HCl (pH 9.0), 2 mM dithiothreitol (DTT)]. Reactions were performed at 30°C for 30 min in a total volume of 50 *µ*L kinase buffer [50 mM Tris–HCl (pH 8.0), 200 mM NaCl, 50 mM MgCl_2_, 0.1% Nonidet P-40, 1 mM DTT] containing 5 *µ*Ci [γ-^32^P] ATP. Samples were then washed with TNE buffer [20 mM Tris–HCl (pH 8.0), 100 mM NaCl, 1 mM EDTA) three times, and the phosphorylated proteins separated by resolution on 8% SDS-PAGE gels. Proteins were stained with CBB, dried, and exposed to X-ray film [43], [44]. To calculate substrate phosphorylation activity, Histone II_AS_ (Sigma-Aldrich) was used as a substrate. To evaluate the effects of PKA-specific inhibitor on histone phosphorylation by TgPKA-C, GST-TgPKA-C and Histone II_AS_ were incubated with 400, 200, 100, 50 and 25 *µ*M H89 (Promega) or 100 *µ*M bovine PKI_5–24_ (synthetic peptide derived from the active domain of PKI; Promega). TgPKA-C activity was measured by scintillation counting of pieces of dried gel corresponding to bands of histone in triplicate.

### Phosphatase treatment

Following the kinase assays, labeled GST fusion proteins were boiled to inactivate the kinase and incubated with λ-protein phosphatase (New England Biolabs) in accordance with the manufacturer's instructions.

### Generation of GFP and TgPKA-R expressing parasites

Transfection of the expression plasmid to *T. gondii* RH/ht^−^ was performed as described previously [45]. Briefly, 30 *µ*g of plasmid DNA was electroporated into 5.0×10^7^ parasites using a GenePulser (Bio-Rad Laboratories, Tokyo, Japan) at 2000 V, 25 *µ*F, and 50 ohm. At 24 h after transfection, parasites were selected by exposure to 50 *µ*g/mL mycophenolic acid and xanthine for 48 h, followed by a subsequent plaque purification. The recombinant clones stably expressing GFP or TgPKA-R-3xFlag were designated RH/GFP or RH/TgPKA-R.

### Parasite growth assay

RH/TgPKA-R or RH/GFP was inoculated onto confluent Vero cell monolayers growing in four-chambered coverglass slides (Nalge Nunc International, Rochester, NY) at a multiplicity of infection (MOI) of 10 and allowed to invade host cells for 2 h at 37°C. Extracellular parasites were then removed by washing three times with PBS(–) and returned to culture in complete medium with or without the PKA-specific inhibitor. The final concentration of inhibitors H89 (Promega) and PKI_14–22_ (Calbiochem, Temecula, CA) was 2 *µ*M, and the volume of DMSO added was the same as that of H89. After incubation for 12, 24, and 36 h, the coverglass slides of RH/TgPKA-R were fixed with 4% paraformaldehyde in PBS(–) for 10 min, permeabilized by 0.1% Triton X-100 in PBS(–) for 10 min, and blocked in 3% bovine serum albumin and 5% skim milk in PBS(–). For the immunofluorescence assay (IFA), the slides were incubated with mouse α-SAG1 (tachyzoite-specific surface antigen) polyclonal antibody (HyTest Ltd., Turku, Finland) at a 1∶1000 dilution for 1 h at room temperature. After reaction with the primary antibody, slides were washed three times with T-PBS [0.1% Tween 20 in PBS(–)], and primary antibody was detected with Alexa 546-conjugated α-mouse antibody (Invitrogen) at a 1∶2000 dilution for 1 h at room temperature, followed by five washes with T-PBS. The coverglass slides of RH/GFP were fixed with 4% paraformaldehyde in PBS(–) for 10 min and then washed three times with PBS(–). Each coverglass slide was dried at room temperature, mounted under a coverglass using Fluoromount-G (SouthernBiotech, Birmingham, AL), and observed under a fluorescence microscope (Olympus, Tokyo, Japan). Microscopic fields were chosen randomly to count parasitophorous vacuoles up to three hundred. Vacuoles were sorted by the numbers of tachyzoites inside, and average numbers of tachyzoites per vacuole were calculated. Parasite growth was determined by the number of parasites per vacuole. The representative data of several experiments are shown in Figure 5.
